# ABO Blood Groups and Incidence of COVID-19 in the Mass Gathering Events in Borriana (Spain), March 2020: A Retrospective Cohort Study

**DOI:** 10.3390/epidemiologia4010007

**Published:** 2023-01-28

**Authors:** Salvador Domènech-Montoliu, Joan Puig-Barberà, Olga Guerra-Murcia, María Rosario Pac-Sa, Alejandro Orrico-Sanchéz, Lorna Gómez-Lanas, Diego Sala-Trull, Carmen Domènech-Leon, Alba Del Rio-González, Manuel Sánchez-Urbano, Paloma Satorres-Martinez, Marta Latorre-Poveda, Sara Ferrando-Rubert, Laura Aparisi-Esteve, Gema Badenes-Marques, Roser Blasco-Gari, Juan Casanova-Suarez, María Fontal-Carcel, María Gil-Fortuño, Noelia Hernández-Pérez, David Jovani-Sales, Laura López-Diago, Cristina Notari-Rodríguez, Oscar Pérez-Olaso, María Angeles Romeu-Garcia, Raquel Ruíz-Puig, Alberto Arnedo-Pena

**Affiliations:** 1 Emergency Service University Hospital de la Plana, 12540 Vila-real, Spain; 2 Vaccines Research Unit, Fundación Para el Fomento de la Investigación Sanitaria y Biomédica de la Comunitat Valenciana, FISABIO-Public Health, 46020 Valencia, Spain; 3 Public Health Center, 12003 Castelló de la Plana, Spain; 4 Epidemiology and Public Health (CIBERESP), 28029 Madrid, Spain; 5 Universidad Católica de Valencia San Vicente Mártir, 46001 Valencia, Spain; 6 Universidad CEU Cardenal Herrera, 12006 Castelló de la Plana, Spain; 7 Health Centers I and II, 12530 Borriana, Spain; 8 Carinyena Health Center, 12540 Vila-real, Spain; 9 Nursing Service University Hospital de la Plana, 12548 Vila-real, Spain; 10 Health Center, 12600 La Vall d’Uixó, Spain; 11 Microbiology Service University Hospital de la Plana, 12540 Vila-real, Spain; 12 Clinical Analysis Service University Hospital de la Plana, 12540 Vila-real, Spain; 13 Department of Health Science, Public University Navarra, 31006 Pamplona, Spain

**Keywords:** COVID-19, SARS-CoV-2, ABO blood groups, incidence, cohort study, mass-gathering events

## Abstract

Our objective was to estimate the incidence of COVID-19 and the ABO blood Groups in the mass-gathering events (MGEs) during the Falles Festival in Borriana (Spain) from 6–10 March 2020. We conducted a population-based retrospective cohort study and measured anti-SARS-CoV-2 antibodies and the ABO of participants. We performed laboratory COVID-19 tests and obtained the ABO in 775 subjects (72.8% of the original exposed cohort): O-group (45.2%), A-group (43.1%), B-group (8.5%) and AB-group (3.4%). Adjusted for confounding factors, including COVID-19 exposure during the MGEs, attack rates of COVID-19 for each ABO group were 55.4%, 59.6%, 60.2%, and 63.7%. The adjusted relative risks were for O-group 0.93 (95% Confidence Interval [CI] 0.83–1.04), for A-group 1.06 (95% CI 0.94–1.18), for B-group 1.04 (95%CI 0.88–1.24), and for AB-group 1.11 (95% CI 0.81–1.51) with no significant differences. Conclusions: Our results suggest no effect of ABO on COVID-19 incidence. We observed weak but not significant protection of the O-group and not a significantly greater infection risk for the remaining groups compared with the O-group. More studies are needed to resolve the controversies regarding the association between ABO and COVID-19.

## 1. Introduction

Like other COVID-19 risk factors, ABO blood groups (ABO) have attracted considerable interest [1,2,3], with a potential role in the stratification of the SARS-CoV-2 infection [4]. Early studies in this field pointed out that O-group subjects experienced a lower risk of infection compared to the other ABOs [5,6,7]. Still, some studies [8,9,10,11] with solid epidemiological designs have not corroborated this differential ABO risk.

In general, studies using blood donors as a control group have found the O-group as protective and the A-group as a risk factor for SARS-CoV-2 infection. However, others have estimated a higher SARS-CoV-2 risk in subjects with B or AB groups [12]. On the other hand, no associations were described among hospitalizations, severity, and death of COVID-19 cases and ABO [13,14,15,16,17,18], but some exceptions have been reported [19,20].

Discordant results have been published on the association between sequelae and complications post-COVID-19 and ABO, with the B-group [21], O-group [22], or AB-group [23] associated with more risk of complications.

There are also discordant results regarding the relationship between the ABO blood groups and COVID-19 incidence. Some authors attributed these discrepancies to a lack of representativeness of cases and controls and selection bias [8,11,24,25].

Correct measurement of SARS-CoV-2 risk exposures in cases and controls plays an essential role in avoiding of selection bias when we try to study a possible association between ABO and COVID-19 incidence. However, risk exposures are usually unmeasured [26,27,28]. In the few studies in which the characteristics of SARS-CoV-2 risk exposures were described, when estimating COVID-19 incidence rates, those were mass-gathering events (MGEs) [21], aircraft carrier crew [10], or household contacts [29]; in each, a protective association with O-group was not found. However, a household contact study reported an enhanced infection risk in A-group subjects [30].

During March 2020, during the *Falles* Festival in Borriana (Spain), several MGEs took place, resulting in a COVID-19 outbreak with 468 cases and a high attack rate of 44.0% [31]. The World Health Organization has defined the MGEs as “events characterized by the concentration of people at a specific location for a particular purpose over a set period of time that have the potential to strain the planning and resources of the host country or community” [32]. We aim to estimate ABO as a potential risk factor for COVID-19 incidence during the indicated MGEs.

## 2. Materials and Methods

We performed a population-based retrospective cohort study of the COVID-19 outbreak in the MGEs of the *Falles* Festival of Borrriana from 6 March to 10 March 2020 by the Public Health Center of Castellon (PHC) and the Emergency Service of the University Hospital de la Plana (UHP) in Vila-real, with 1064 participants with laboratory COVID-19 tests performed, resulting 468 cases and 596 no-cases. The methods of this study were detailed in a previous publication [31]. The participants were a representative sample of the 2800 members from 19 “*falla*”associations organizing the *Falles* Festival in Borriana. We carried a simple random sampling, with 19 clusters, one per “*falla*”, plus a random sampling of the 400 attendees to the Queen’s gala dinner. As a result we sampled 1663 individuals, and finally, 1338 gave their consent to be enrolled. From this population, the total of 1064 participants could be suffering from the disease or remained uninfected in the period from 6 March to 31 March, considering a maximum incubation period of 14 days.

**Figure 1:**
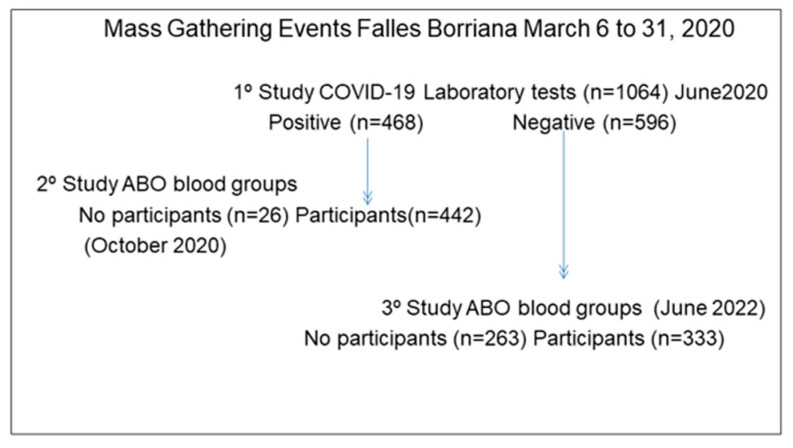
Flow chart of the three studies of COVID-19 and ABO blood groups in the Borriana COVID-19 cohort.

We have conducted three follow-up studies of this cohort: the first in June 2020 [31], the second in October 2020 [21], and the last in June 2022. In the first study, COVID-19 infection was determined by anti-SARS-CoV-2 nucleocapsid protein N antibodies in participants with electrochemiluminescence immunoassay [33] (Elecsys^®^, Mannheim, Germany, Anti-SARS-CoV-2, Roche Diagnostics), a reverse transcription polymerase chain reaction (RT-PRC) [34] (LightMix^®^ Modular Sarbecovirus E-gene with the LightCycler^®^ 480 II system Roche, Basel, Switzerland) or rapid tests antigen in 11 participants [35] (Healgen Scientific LLC for COVID-19 IgG/IgM rapid test cassette) Houston, Texas, USA). The Clinical Analysis and Microbiology Service of UHP and other public and private laboratories performed these determinations. The second study was carried out only in the participants positive for SARS-CoV-2 infection, and ABO blood groups were determined in 442 subjects. Finally, in the third study, we included positive and non-positive subjects, and ABO was determined to complete this information in all participants. Then, 775 subjects had their ABO analyzed (Figure 1). The ABO blood group was analyzed in the Hematology Service of the HUP by the gel test [36] (ID-Card ABO/RhD, DiaMed GmbH, Bio-Rad Laboratories Switzerland). Finally, the ABO groups were obtained in 94.4% (442/468) of positive SARS-CoV-2 subjects and in 55.9% (333/596) of negative SARS-CoV-2 subjects.

In addition to the serologic and blood studies, a telephone questionnaire was administered in each of the three follow-up studies to complete information on COVID-19 exposure in MGEs and families, socio demographic characteristics, habits and health status, body mass index (BMI), and underlying conditions. The questionnaire was administered by health staff of the health centers of Borriana, Vila-real, Onda, Vall d’Uixo, the Emergency Service of UHP, and the PHC. For the present study, only the questionnaire from the first study was used.

### Statistical Methods

We estimated the mean and their standard deviation, or percentages, to report the characteristics of the participants considering the ABO status and the SARS-CoV-2 infection outcome. For comparisons among qualitative variables, we used the Chi2 and the Fisher exact tests, and for quantitative variables, the Kruskal–Wallis test. We estimated the crude attack rates (cAR) for each ABO blood group by dividing the positive SARS-CoV-2 infection by the total in each group. We used Poisson regression to estimate crude relative risk (cRR) of SARS-CoV-2 infection among the ABO blood groups and their 95% confidence interval (CI). We used Direct Acyclic Graph [37] and the DAGitty 3.0 program (Johannes Textor, Nijmegen, The Netherlands) [38] to identify confounding. The adjusted factors were the following: age, sex, BMI, smoking habit, falla, social class (upper and middle versus lower class, chronic disease, and COVID-19 exposures (numbers of MGEs, family COVID-19 cases, contact with COVID-19 cases, and observed a person with cough or cold at the MGEs. Social class was assigned according to occupation [39]; from the occupation group I and II, professional and technical occupations (codified as upper and middle class), and groups III–VI, skilled, non-manual or manual, semi-skilled, and unskilled occupations (codified as a lower class).

Finally, we used an inverse probability-weighted regression to estimate the adjusted attack rate (aAR) and adjusted relative risk (aRR) [40]. In addition, we performed a sensitivity analysis in the asymptomatic SARS-CoV-2 infections in the first study with a total of 57 subjects, 85% of the 67 asymptomatic SARS-CoV-2 infections [31], considering that asymptomatic cases could have protection against the disease and that it may be related to ABO; fewer SARS-CoV-2 infections in asymptomatic O-group subjects have been reported [41]. We performed all statistical analysis using STATA^®^ 14.2 version (StataCorp. College Station, TX, USA). The significant level for comparisons was *p* ≤ 0.05.

This study was approved by the Ethics Committee of the University Hospital de la Plana in Vila-real. All participants or the parents of minors provided written informed consent.

## 3. Results

We enrolled 775 subjects in this study, with a participation rate of 72.6%, considering 1064 participants with laboratory test determinations in the first study of the *Fallas* Festival in the Borriana COVID-19 outbreak.

The characteristics of participants by ABO are shown in Table 1. Among the 775 members of the cohort, the distribution of ABO was as follows: O (45.3%), A (43.1%), B (8.5%), and AB (3.4%). The mean age in years varied from 36.4 to 38.9, and the female percentage was between 41.7% (AB-group) and 68.2% (B-group). Asymptomatic cases went from 4.6% (B-group) to 9.0% (A-group). Social class, obesity, BMI, smoking, and chronic disease prevalence were similar among the four groups. Exposure to COVID-19 cases was higher in the A-group, with significant differences in the affirmative. The mean age of symptomatic cases was 38.4 ± 16.5 years, and the mean age of asymptomatic cases was 29.5 ± 18.3 years (*p* = 0.0002).

Table 2 shows the association between ABO and COVID-19 incidence. Crude attack rates varied from 53.0% in O-group to 62.5% in AB-group, with no significant differences between groups. When the O-group subjects were compared with the Non-O-group subjects, we found no significant differences in SARS-CoV-2 infection (cRR = 0.88 95% CI 0.73–1.06). Similarly, we found no significant differences when comparing the A with the No-A group subjects (cRR = 1.09 95% CI 0.90–1.32), the B with the No-B group (cRR = 1.10 95% CI 0.80-1.51), and the AB with the No-AB group subjects (cRR = 1.10 95% CI 0.66–1.84).

Adjusted attack rates varied from 55.4% (O-group) to 63.7% (AB-group) (Table 2). The O-group had fewer SARS-CoV-2 infections. However, it was not significant when compared with the Non-O groups (aRR = 0.93 95% CI 0.83–1.04). Comparisons of the remaining groups did not provide significant results either: the A-group with the Non-A-group (aRR = 1.06 95% CI 0.94–1.18), the B-group with the Non-B-group (aRR = 1.04 95% CI 0.88–1.24), and the AB-group with the Non-AB-groups (aRR = 1.11 95% CI 0.81–1.51). Taking the O-group as a reference, the remaining groups had higher but not significant crude and adjusted attack rates.

As a sensitivity analysis, we estimated the crude and adjusted SARS-CoV-2 attack rates by ABO groups, restricted to the 57 asymptomatic SARS-CoV-2 infections ascertained in the first study (Table 3). When comparing each group with the remaining groups, the cAR varied from 10.7% (B-group) to 18.3% (A-group), and the cRR from 0.68 (95% CI 0.40–1.16), for the O-group to 1.53 (95% CI 0.91–2.58), and for the A-group there were no significant differences. The adjusted relative risk varied from 0.03 (95% CI 0.00–10.70) for the B-group to 1.59 (95% CI 0.98–2.61) for the A-group, but we could discard attack rate homogeneity as all comparisons were non-significant. When we compared with the O-group as a reference group, the differences were not significant. The A-group had a higher adjusted attack rate than the O-group, with an adjusted relative risk of 1.49 (95% CI 0.90–2.44).

## 4. Discussion

The results suggest that the differential risk of subjects in the O-group against SARS-CoV-2 infection was modest and insufficient to obtain significant differences when the SARS-CoV-2 exposure of each participant was measured and adjusted for potential confounders. In addition, the other Non-O groups presented a small risk of SARS-CoV-2 infection but, again, without significant differences. Following Monson’s scale of RR strength, we did not detect an effect of the ABO blood groups on SARS-CoV-2 infection, as all aRR were in the range of 0.90–1.20 [42]. For asymptomatic cases, the A-group presented a moderate association with asymptomatic SARS-CoV-2 infection, aRR range of 1.30–3.0 on Monson’s scale. The aRR and the cRR had similar magnitude, suggesting that the potential confounder effects were limited. In addition, asymptomatic SARS-CoV-2 infections were more frequent in the A-group, considered the higher-risk group in some studies. This finding was not consistent with other studies [43,44,45], but the others have also found no differences among ABO [46]. On the other hand, in our second study [21], with only COVID-19 cases compared with an ABO distribution in an active general population of a Spanish Mediterranean zone, no significant differences in COVID-19 incidence among ABO were found [47].

Our results are in line with several studies, including prospective cohort studies [10,29], case-control studies [9,48], and cross-sectional studies [8,49,50], where control groups were obtained from an exposed population, admissions, or patients tested for SARS-CoV-2, households cases, and blood donors. Studies on asymptomatic blood donors and SARS-CoV-2 infection have reported contradictory results; in some cases, these associations were not found [11,51,52,53], and in others, reporting that the O-group was protective [28,41,44].

In general, higher protection of the O-group or higher risk of the A-group were found in studies that used blood donors, who, as universal donors, are overrepresented in samples gathered from populations with ABO known for different causes and diseases [54,55,56]. In addition, no control of potential confounders was performed in some studies [57]. In a systematic review of 24 studies of ABO and COVID-19, Bai and co-authors [58] found several methodological problems in many studies, including selection bias, and the control of confounding suggested no association. When controlling for race and ethnicity, no difference in risk was detected in a study in Colorado (USA) [59]. However, genetic associations with both the risk of infection and disease severity have been reported for the ABO locus, with the O-group being protective when compared with the other ABO groups [60]. In addition, no A-groups could be less susceptible due to the production of anti-A antibodies [61]. Low ABO antibody levels have been found in COVID-19 cases compared with controls, suggesting that ABO antibodies play a role in protecting against the infection [62]. However, no relevant neutralizing activity in ABO has been found in another study [63], and the levels of SARS-CoV-2 neutralizing antibodies were also not significantly associated with the ABO Rh(D) group [50]. On the other hand, there are a total of 43 blood group systems with 345 distinct antigen specificities with variations in human populations [64] and only four basic ABO groups. It is suggested that the high simplification of the comparisons could result in residual confounding [8].

In general, meta-analysis studies on ABO groups and SARS-CoV-2 infection report a high risk for the A-group and a lower risk for the O-group [43,65], but these studies present differences in design, sample size, representativeness, exposures, and in some cases, significant heterogeneity [1,66]. In 2021, a study by the International Society of Blood Transfusion (ISBT) COVID-19 Working Group [67] concluded “that prospective and mechanistic studies are needed to verify several of the proposed associations between the ABO groups and COVID-19 risk of infection. Based on the strength of available studies, there are insufficient data for guiding policy in this regard”. Conversely, on the other hand, higher mortality associated with the A and the B groups has been reported [68].

With respect to transmission, if the O-group has some infection protection, this protection is overcome considering the Ellis model of ABO incompatibility [69]; the Non-O groups could experience a higher infection incidence, but when the majority of the population is infected, the O-group suffers similar infection rates.

The biological aspect of the relationship between ABO and COVID-19 infection has several elements [3], including the anti-A antibodies, production of glycan antigens by the SARS-CoV-2, coagulation system, and genetic variations in the ABO gene. Various mechanisms have been proposed for the association between the ABO blood type and SARS-CoV-2 infection [66]. Considering the three major hypotheses [3], SARS-CoV-2 with ABO (H)-like structures would transmit in different grades due to the presence of ABO antibodies, ABH antigens could enhance SARS-CoV-2 invasion, and the risk of thromboembolic events in Non-O groups could give some protection of O-group against of severe outcomes. However, experimental data to confirm these hypotheses is non-existent [2].

The strengths of our study reside in the cohort design, adjustment for exposure and other potential confounding factors, the representative sample of the *Fallas* population exposed to MGEs, with elevated participation, and the power of this study could arrive at 86%. In addition, anti-SARS-CoV-2 antibodies were used to test most participants with a highly sensitive and specific laboratory technique.

This study has several limitations. First, SARS-CoV-2 genetic variants were not identified. Second, exposure was measured retrospectively and may be subjected to a recall bias. We think that this bias may be limited in our study, considering that this bias is independent of the ABO group of each participant. However, remembering that the exposition could be associated with the disease, and cases, therefore, may better remember the exposition, which could bias the effect of exposition factors. Third, we did not consider the complications of COVID-19. Fourth, COVID-19 is a new disease, and variables not included could be potential confounders. Fifth, the participation rate of COVID-19 cases was higher than no-cases, and some ascertainment bias may happen if ABO groups have different participation rates, but the distribution of ABO in our study was not different from the reported ABO distribution of the general population of the Spanish Mediterranean zone [47] (*p* = 0.484), suggesting that this bias may be minor. Finally, regarding the sensitivity analysis, the number of asymptomatic cases was too small to obtain robust conclusions.

Considering the controversial issues among studies, conducting prospective cohort studies in different geographic areas, including the study of virus variants, genetic background, and potential confounders, is needed to establish the effects of ABO on COVID-19 incidence.

## 5. Conclusions

Our results suggest that there is no association of ABO with COVID-19 incidence. We observed a weak but not significant protection of the O-group and no significantly higher COVID-19 risk in the remaining groups compared with the O-group. More epidemiologic studies of ABO and COVID-19 incidence could be necessary to resolve current inconsistencies in the potential COVID-19-related risk due to ABO.

## Figures and Tables

**Table 1:** Characteristics of ABO blood groups. Borriana COVID-19 cohort, 2020.

	Blood Group Distribution				
Variables	0 N (%)	A N (%)	B N (%)	AB N (%)	*p*-Value
N = 775	351 (45.3)	334 (43.1)	66 (8.5)	24 (3.1)	-
Female	219 (62.4)	197 (59.0)	47 (68.2)	14 (41.7)	0.114
Age (years) mean ± SD ^1^	37.1 ± 18.0	38.9 ± 16.6	36.4 ± 18.1	37.2 ± 14.6	0.861
Asymptomatic cases	22 (6.3)	30 (9.0)	3 (4.6)	2 (8.3)	0.445
Social class I–II ^2,3^	97 (27.9)	84 (25.2)	22 (33.3)	5 (20.8)	0.487
Social class III–VI	251 (72.1)	250 (74.8)	44 (66.7)	19 (79.2)	
BMI mean ± SD ^4^	24.5 ± 5.2	25.3 ± 5.0	24.0 ± 5.3	25.0 ± 5.1	0.133
BMI ≥ 30 kg/m 2	53 (15.3)	57 (17.2)	9 (13.6)	3 (12.5)	0.809
Current smoker ^5^	65 (18.6)	70 (21.0)	16 (24.2)	1 (4.2)	0.277
Ex-smoker	74 (21.2)	68 (20.4)	10 (15.2)	8 (33.3)	
No smoker	210 (60.2)	195 (58.6)	40 (60.6)	15 (62.5)	
Chronic disease ^6^	124 (35.4)	116 (35.0)	23 (34.8)	7 (29.2)	0.959
Exposure to COVID-19					
Mass-gathering events (MGEs)	1.6 ± 1.2	1.7 ± 1.3	1.7 ± 1.0	1.8 ± 1.4	0.788
Family COVID-19 case ^7^	176 (50.9)	167 (50.2)	41 (63.1)	11 (45.8)	0.249
Contact COVID-19 case ^8^	254 (74.3)	254 (77.2)	47 (72.3)	13 (54.2)	0.104
Observed a person with cough ^9^ or cold at MGEs	130 (37.7)	130 (39.6)	17 (26.2)	4 (16.7)	0.033

^1^ SD = standard deviation. ^2^ Social class I–II. ^3^ Missing in three participants. ^4^ Missing in nine participants. ^5^ Missing in three participants. ^6^ Missing in four participants. ^7^ Missing in seven participants. ^8^ Missing in 15 participants. ^9^ Missing in 13 participants.

**Table 2:** Incidence of COVID-19 cases by ABO blood groups. Inverse probability-weighted regression. Crude attack rate (cAR) and adjusted (aAR). Crude relative risk (cRR) and adjusted (aRR); 95% confidence interval (CI). Borriana COVID-19 cohort, 2020.

Blood Groups	Cases	No-Cases	cAR(%)	cRR (95% CI)	aAR (%)	aRR ^1^ (95% CI)	*p*-Value
Each group compared with the other groups							
O	186	165	53.0	0.88 (0.73–1.06)	55.4	0.93 (0.83–1.04)	0.209
No-O	256	168	60.4	1.00	59.7	1.00	
A	200	134	59.9	1.09 (0.90–1.32)	59.6	1.06 (0.94–1.18)	0.341
No-A	242	199	54.9	1.00	56.4	1.00	
B	41	25	62.1	1.10 (0.80–1.51)	60.2	1.04 (0.88–1.24)	0.628
No-B	401	308	56.6	1.00	57.7	1.00	
AB	15	9	62.5	1.10 (0.66–1.84)	63.7	1.11 (0.81–1.51)	0.518
No-AB	427	324	56.9	1.00	57.5	1.00	
O-group as the base for comparison							
O	186	165	53.0	1.00	55.4	1.00	
A	200	134	59.9	1.12 (0.93-1.38)	59.5	1.07 (0.61–1.21)	0.246
B	41	25	62.1	1.17 (0.84–1.64)	60.2	1.09 (0.90–1.31)	0.383
AB	15	9	62.5	1.18 (0.70–2.00)	63.6	1.15 (0.84–1.57)	0.397

^1^ Adjusted for age, sex, BMI, smoking habit, *falla*, social class, chronic disease, COVID-19 exposures.

**Table 3:** Asymptomatic SARS-CoV-2 infections by ABO blood groups. Crude attack rate (cAR) and adjusted (aAR). Crude relative risk (cRR) and adjusted (aRR); 95% confidence interval (CI). Borriana COVID-19 Cohort, 2020.

Blood Groups	Cases N(%) ^1^	No-Cases	cAR(%)	cRR (95% CI)	aAR(%)	aRR ^2^ (95% CI)	*p*-Value
Each group compared with the other groups							
0	22 (10.7)	165	11.8	0.68 (0.40–1.16)	12.3	0.75 (0.46–1.24)	0.264
No-O	35	168	17.2	1.00	16.3	1.00	
A	30 (12.8)	134	18.3	1.53 (0.91–2.58)	18.3	1.59 (0.98–2.61)	0.062
No-A	27	199	12.0	1.00	11.5	1.00	
B	3 (6.7)	25	10.7	0.72 (0.22–2.30)	0.4	0.03 (0.00–10.70)	0.233
No-B	54	308	14.9	1.00	15.8	1.00	
AB	2 (10.5)	9	18.2	1.25 (0.31–5.13)	NC^3^		
No-AB	55	324	14.5	1.00			
O-group as the base for comparison							
O	22	165	11.8	1.00	12.4	1.00	
A	30	134	18.3	1.55 (0.90–2.70)	18.4	1.49 (0.90–2.44)	0.119
B	3	25	10.7	0.91 (0.27–3.04)	0.4	0.03 (0.00–10.59)	0.247
AB	2	9	18.2	1.55 (0.36–6.57)	NC^3^		

^1^ Percentage of the total of COVID-19 cases per ABO groups. ^2^ Adjusted for age, sex, BMI, smoking habit, *falla*, social class, chronic disease, COVID-19 exposures. ^3^ Not calculable.

## Data Availability

Data from this study can be consulted if the authors are requested.

## References

[1] Liu N., Zhang T., Ma L., Zhang H., Wang H., Wei W., Pei H., Li H. (2021). The impact of ABO blood group on COVID-19 infection risk and mortality: A systematic review and meta-analysis. Blood Rev..

[2] Bullerdiek J., Reisinger E., Rommel B., Dotzauer A. (2022). ABO blood groups and the risk of SARS-CoV-2 infection. Protoplasma.

[3] Pereira E., Felipe S., de Freitas R., Araújo V., Soares P., Ribeiro J., Henrique Dos Santos L., Alves J.O., Canabrava N., van Tilburg M. (2022). ABO blood group and link to COVID-19: A comprehensive review of the reported associations and their possible underlying mechanisms. Microb. Pathog..

[4] Pendu J.L., Breiman A., Rocher J., Dion M., Ruvoën-Clouet N. (2021). ABO blood types and COVID-19: Spurious, anecdotal, or truly important relationships? A reasoned review of available data. Viruses.

[5] Zhao J., Yang Y., Huang H., Li D., Gu D., Lu X., Zhang Z., Liu L., Liu T., Liu Y. (2021). Relationship Between the ABO Blood Group and the Coronavirus Disease 2019 (COVID-19) Susceptibility. Clin. Infect. Dis..

[6] Barnkob M.B., Pottegård A., Støvring H., Haunstrup T.M., Homburg K., Larsen R., Hansen M.B., Titlestad K., Aagaard B., Møller B.K. (2020). Reduced prevalence of SARS-CoV-2 infection in ABO blood group O. Blood Adv..

[7] Zietz M., Zucker J., Tatonetti N.P. (2020). Associations between blood type and COVID-19 infection, intubation, and death. Nat. Commun..

[8] Dzik S., Eliason K., Morris E.B., Kaufman R.M., North C.M. (2020). COVID-19 and ABO blood groups. Transfusion.

[9] Anderson J.L., May H.T., Knight S., Bair T.L., Muhlestein J.B., Knowlton K.U., Horne B.D. (2021). Association of sociodemographic factors and blood group type with risk of COVID-19 in a US population. JAMA Netw. Open.

[10] Boudin L., Janvier F., Bylicki O., Dutasta F. (2020). ABO blood groups are not associated with risk of acquiring the SARS-CoV-2 infection in young adults. Haematologica.

[11] Focosi D., Carla I.M., Lanza M. (2021). ABO blood group correlations with Covid-19: Cohort choice makes a difference. Clin. Infect. Dis..

[12] Matzhold E.M., Berghold A., Bemelmans M.K.B., Banfi C., Stelzl E., Kessler H.H., Steinmetz I., Krause R., Wurzer H., Schlenke P. (2021). Lewis and ABO histo-blood types and the secretor status of patients hospitalized with COVID-19 implicate a role for ABO antibodies in susceptibility to infection with SARS-CoV-2. Transfusion.

[13] Latz C.A., DeCarlo C., Boitano L., Png C.Y.M., Patell R., Conrad M.F., Eagleton M., Dua A. (2020). Blood type and outcomes in patients with COVID-19. Ann. Hematol..

[14] Akhlaq H., Tizro P., Aggarwal A., Nava V.E. (2022). ABO blood group association with COVID-19 mortality. J. Hematol..

[15] Ishaq U., Malik A., Malik J., Mehmood A., Qureshi A., Laique T., Zaidi S.M.J., Javaid M., Rana A.S. (2021). Association of ABO blood group with COVID-19 severity, acute phase reactants and mortality. PLoS ONE.

[16] Lehrer S., Rheinstein P.H. (2021). ABO blood groups, COVID-19 infection and mortality. Blood Cells Mol. Dis..

[17] Mullins J., Al-Tarbsheh A.H., Chieng H., Chaukiyal P., Ghalib S., Jain E., Dawani O., Santelises Robledo F.M., Chong W.H., Feustel P.J. (2021). The association of ABO blood type with the risk and severity of COVID-19 infection. Am. J. Blood Res..

[18] Kabrah S.M., Abuzerr S.S., Baghdadi M.A., Kabrah A.M., Flemban A.F., Bahwerth F.S., Assaggaf H.M., Alanazi E.A., Alhifany A.A., Al-Shareef S.A. (2021). Susceptibility of ABO blood group to COVID-19 infections: Clinico-hematological, radiological, and complications analysis. Medicine.

[19] Ayatollahi A.A., Aghcheli B., Amini A., Nikbakht H., Ghassemzadehpirsala P., Behboudi E., Rajabi A., Tahamtan A. (2021). Association between blood groups and COVID-19 outcome in Iranian patients. Future Virol..

[20] Muñiz-Diaz E., Llopis J., Parra R., Roig I., Ferrer G., Grifols J., Millán A., Ene G., Ramiro L., Maglio L. (2021). Relationship between the ABO blood group and COVID-19 susceptibility, severity and mortality in two cohorts of patients. Blood Transfus..

[21] Domènech-Montoliu S., Puig-Barberà J., Pac-Sa M.R., Vidal-Utrillas P., Latorre-Poveda M., Rio-González A.D., Ferrando-Rubert S., Ferrer-Abad G., Sánchez-Urbano M., Aparisi-Esteve L. (2021). ABO blood groups and the incidence of complications in COVID-19 patients: A population-based prospective cohort study. Int. J. Environ. Res. Public Health.

[22] Ghamdi F.A., Naqvi S., Alabassi F.A., Alhayyani S., Baig M.R., Kumar V., Anwar F. (2022). Alterations in clinical characteristics of blood donors post COVID-19 recovery. Curr. Pharm. Des..

[23] Nafakhi A., Rabeea I.S., Al-Darraji R., Nafakhi H., Mechi A., Al-Khalidi A., Alareedh M. (2022). Association of ABO blood group with in-hospital adverse outcome and long term persistent symptoms of COVID-19 infection: A single-center longitudinal observational study. Health Sci. Rep..

[24] Griffith G.J., Morris T.T., Tudball M.J., Herbert A., Mancano G., Pike L., Sharp G.C., Sterne J., Palmer T.M., Davey Smith G. (2020). Collider bias undermines our understanding of COVID-19 disease risk and severity. Nat. Commun..

[25] Boudin L., Dutasta F. (2021). Relationship between ABO blood groups and coronavirus Disease 2019: Study design matters. Clin. Infect. Dis..

[26] Chegni H., Pakravan N., Saadati M., Ghaffari A.D., Shirzad H., Hassan Z.M. (2020). Is there a link between COVID-19 mortality with genus, age, ABO blood group type, and ACE2 gene polymorphism?. Iran J. Public Health.

[27] Gurung S., Mahotra N.B., Shrestha L., Sherpali A., Joshi S.P., Shrestha G., Shrestha S., Shakya A., Kandel M. (2022). Association of ABO blood group with susceptibility to SARS-CoV-2 infection in Rupandehi district of Nepal. SAGE Open Med..

[28] Solmaz İ., Araç S. (2021). ABO blood groups in COVID-19 patients; Cross-sectional study. Int. J. Clin. Pract..

[29] Janda A., Engel C., Remppis J., Enkel S., Peter A., Hörber S., Ganzenmueller T., Schober S., Weinstock C., Jacobsen E.M. (2022). Role of ABO blood group in SARS-CoV-2 infection in households. Front. Microbiol..

[30] Boukhari R., Breiman A., Jazat J., Ruvoën-Clouet N., Martinez S., Damais-Cepitelli A., Le Niger C., Devie-Hubert I., Penasse F., Mauriere D. (2022). ABO blood group incompatibility protects against SARS-CoV-2 transmission. Front. Microbiol..

[31] Domènech-Montoliu S., Pac-Sa M.R., Vidal-Utrillas P., Latorre-Poveda M., Del Rio-González A., Ferrando-Rubert S., Ferrer-Abad G., Sánchez-Urbano M., Aparisi-Esteve L., Badenes-Marques G. (2021). Mass gathering events and COVID-19 transmission in Borriana (Spain): A retrospective cohort study. PLoS ONE.

[32] World Health Organizarion (2015). Public Health for Mass Gatherings: Key Considerations Iinterim Guidamce. Ginebre. https://www.who.int/publications/i/item/public-health-for-mass-gatherings-key-considerations.

[33] Egger M., Bundschuh C., Wiesinger K., Gabriel C., Clodi M., Mueller T., Dieplinger B. (2020). Comparison of the Elecsys^®^ Anti-SARS-CoV-2 immunoassay with the EDI™ enzyme linked immunosorbent assays for the detection of SARS-CoV-2 antibodies in human plasma. Clin. Chim. Acta.

[34] Yip C.C., Sridhar S., Cheng A.K., Leung K.H., Choi G.K., Chen J.H., Poon R.W., Chan K.H., Wu A.K., Chan H.S. (2020). Evaluation of the commercially available LightMix^®^ Modular E-gene kit using clinical and proficiency testing specimens for SARS-CoV-2 detection. J. Clin. Virol..

[35] Dellière S., Salmona M., Minier M., Gabassi A., Alanio A., Le Goff J., Delaugerre C., Chaix M.L., Saint-Louis CORE (COvid REsearch) Group (2020). Evaluation of the COVID-19 IgG/IgM Rapid Test from Orient Gene Biotech. J. Clin. Microbiol..

[36] Lapierre Y., Rigal D., Adam J., Josef D., Meyer F., Greber S., Drot C. (1990). The gel test: A new way to detect red cell antigen-antibody reactions. Transfusion.

[37] Greenland S., Pearl J., Robins J.M. (1999). Causal diagrams for epidemiologic research. Epidemiology.

[38] Textor J., van der Zander B., Gilthorpe M.S., Liskiewicz M., Ellison G.T. (2016). Robust causal inference using directed acyclic graphs: The R package ‘dagitty’. Int. J. Epidemiol..

[39] Alonso J., Pérez P., Sáez M., Murillo C. (1997). Validez de la ocupación como indicador de la clase social, según la clasificación del British Registrar General. Gac. Sanit..

[40] Robins J.M., Hernán M.A., Brumback B. (2000). Marginal structural models and causal inference in epidemiology. Epidemiology.

[41] Valenti L., Pelusi S., Cherubini A., Bianco C., Ronzoni L., Uceda Renteria S., Coluccio E., Berzuini A., Lombardi A., Terranova L. (2021). Trends and risk factors of SARS-CoV-2 infection in asymptomatic blood donors. Transfusion.

[42] Monson R. (1982). Occupational Epidemiology.

[43] Wu B.B., Gu D.Z., Yu J.N., Yang J., Shen W.Q. (2020). Association between ABO blood groups and COVID-19 infection, severity and demise: A systematic review and meta-analysis. Infect. Genet. Evol..

[44] Singh P.P., Srivastava A.K., Upadhyay S.K., Singh A., Upadhyay S., Kumar P., Rai V., Shrivastava P., Chaubey G., Serosurveillance Consortium BHU (2021). The association of ABO blood group with the asymptomatic COVID-19 cases in India. Transfus. Apher. Sci..

[45] Kotila T.R., Alonge T.O., Fowotade A., Famuyiwa O.I., Akinbile A.S. (2021). Association of the ABO blood group with SARS-CoV-2 infection in a community with low infection rate. Vox. Sang..

[46] Das S.S., Bera S.C., Biswas R.N. (2022). Seroprevalence of anti-severe acute respiratory syndrome coronavirus 2 antibody among healthy blood donors in a hospital-based blood center in Eastern India during the COVID-19 pandemic. Asian J. Transfus. Sci..

[47] Nogareda-Barbudo A. (1964). Grupos sanguineos en la población activa española. An. Med. Cir..

[48] Khalil A., Feghali R., Hassoun M. (2020). The Lebanese COVID-19 Cohort; a challenge for the ABO blood group system. Front. Med..

[49] Levi J.E., Telles P.R., Scrivani H., Campana G. (2021). Lack of association between ABO blood groups and susceptibility to SARS-CoV-2 infection. Vox. Sang..

[50] Hirani R., Hoad V., Gosbell I.B., Irving D.O. (2022). Absence of correlation between ABO Rh(D) blood group and neutralizing antibody titers in SARS-CoV-2 convalescent plasma donors. Transfusion.

[51] Alzabeedi K.H., Makhlof R.T.M., Bakri R.A., Ewis A.A., Alhamdi H.W., Habeebullah T.M.A., Khogeer A.A., Mulla E.A.A., Roshan S.A.M., Qabbani F.H. (2022). High seroprevalence of SARS-CoV-2 IgG and RNA among asymptomatic blood donors in Makkah Region, Saudi Arabia. Vaccines.

[52] Hasan M., Moiz B., Qaiser S., Masood K.I., Ghous Z., Hussain A., Ali N., Simas J.P., Veldhoen M., Alves P. (2022). IgG antibodies to SARS-CoV-2 in asymptomatic blood donors at two time points in Karachi. PLoS ONE.

[53] Kale P., Patel N., Gupta E., Bajpai M. (2022). SARS-Coronavirus-2 seroprevalence in asymptomatic healthy blood donors: Indicator of community spread. Transfus. Apher. Sci..

[54] Ellinghaus D., Degenhardt F., Bujanda L., Buti M., Albillos A., Invernizzi P., Fernández J., Prati D., Baselli G., Severe Covid-19 GWAS Group (2020). Genomewide association study of severe Covid-19 with respiratory failure. N. Engl. J. Med..

[55] Göker H., Aladağ Karakulak E., Demiroğlu H., Ayaz Ceylan Ç.M., Büyükaşik Y., Inkaya A.Ç., Aksu S., Sayinalp N., Haznedaroğlu I.C., Uzun Ö. (2020). The effects of blood group types on the risk of COVID-19 infection and its clinical outcome. Turk. J. Med. Sci..

[56] Enguita-Germán M., Librero J., Leache L., Gutiérrez-Valencia M., Tamayo I., Jericó C., Gorricho J., García-Erce J.A. (2022). Role of the ABO blood group in COVID-19 infection and complications: A population-based study. Transfus. Apher. Sci..

[57] Pandey H.C., Dhiman Y., Chippy S.C., Coshic P., Jain P. (2021). Seroprevalence of SARS-Coronavirus 2 among asymptomatic healthy blood donors from healthcare and non-healthcare settings: Implications for safety of blood donors and blood collection staff during blood donation. Transfus. Apher. Sci..

[58] Bai Y., Yan Z., Murray E.J. Systematic review of the association between ABO blood type and COVID-19 incidence and mortality. medRxiv.

[59] Pasko B.E., Abbott D., Bocsi G.T., Draper N.L. (2022). ABO blood groups Are not associated with COVID-19 disease incidence and severity when correcting for ethnicity differences in blood type. Am. J. Clin. Pathol..

[60] Shelton J.F., Shastri A.J., Ye C., Weldon C.H., Filshtein-Sonmez T., Coker D., Symons A., Esparza-Gordillo J., Aslibekyan S., 23 andMe COVID-19 Team (2021). Trans-ancestry analysis reveals genetic and nongenetic associations with COVID-19 susceptibility and severity. Nat. Genet..

[61] Zhang Y., Garner R., Salehi S., La Rocca M., Duncan D. (2021). Association between ABO blood types and coronavirus disease 2019 (COVID-19), genetic associations, and underlying molecular mechanisms: A literature review of 23 studies. Ann. Hematol..

[62] Deleers M., Breiman A., Daubie V., Maggetto C., Barreau I., Besse T., Clémenceau B., Ruvoën-Clouet N., Fils J.F., Maillart E. (2021). Covid-19 and blood groups: ABO antibody levels may also matter. Int. J. Infect. Dis..

[63] Focosi D., Rosellini A., Spezia P.G., Macera L., Lanza M., Paolicchi A., Biagini D., Baj A., Pistello M., Maggi F. (2021). Lack of neutralizing activity in nonconvalescent sera, regardless of ABO blood group and anti-A isoagglutinin titer. J. Clin. Virol. Plus.

[64] Gassner C., Castilho L., Chen Q., Clausen F.B., Denomme G.A., Flegel W.A., Gleadall N., Hellberg Å., Ji Y., Keller M.A. (2022). International Society of Blood Transfusion Working Party on Red Cell Immunogenetics and Blood Group Terminology Report of Basel and three virtual business meetings: Update on blood group systems. Vox. Sang..

[65] Shibeeb S., Khan A. (2022). ABO blood group association and COVID-19. COVID-19 susceptibility and severity: A review. Hematol. Transfus. Cell Ther..

[66] Gutiérrez-Valencia M., Leache L., Librero J., Jericó C., Enguita-Germán M., García-Erce J.A. (2022). ABO blood group and risk of COVID-19 infection and complications: A systematic review and meta-analysis. Transfusion.

[67] Goel R., Bloch E.M., Pirenne F., Al-Riyami A.Z., Crowe E., Dau L., Land K., Townsend M., Jecko T., Rahimi-Levene N. (2021). ABO blood group and COVID-19: A review on behalf of the ISBT COVID-19 Working Group. Vox. Sang..

[68] Szymanski J., Mohrmann L., Carter J., Nelson R., Chekuri S., Assa A., Spund B., Reyes-Gil M., Uehlinger J., Baron S. (2021). ABO blood type association with SARS-CoV-2 infection mortality: A single-center population in New York City. Transfusion.

[69] Ellis P.J.I. (2021). Modelling suggests ABO histo-incompatibility may substantially reduce SARS-CoV-2 transmission. Epidemics.

